# Intervening and reducing sharing of false cancer treatments on social media: Online experiment

**DOI:** 10.1371/journal.pone.0341907

**Published:** 2026-02-25

**Authors:** Allison J. Lazard, Shelby Lake, Tara Licciardello Queen, Mirian Avendaño-Galdamez, Scott Babwah Brennen, Tushar Varma, Hung-Jui Tan, Marjory Charlot, Nabarun Dasgupta

**Affiliations:** 1 Hussman School of Journalism and Media, University of North Carolina at Chapel Hill, Chapel Hill, North Carolina, United States of America; 2 Lineberger Comprehensive Cancer Center, University of North Carolina at Chapel Hill, Chapel Hill, North Carolina, United States of America; 3 Department of Health Behavior, Gillings School of Global Public Health, University of North Carolina at Chapel Hill, Chapel Hill, North Carolina, United States of America; 4 Center on Technology Policy, New York University, New York, New York, United States of America; 5 Department of Urology, School of Medicine, University of North Carolina at Chapel Hill, Chapel Hill, North Carolina, United States of America; 6 Department of Medicine, Division of Oncology, School of Medicine, University of North Carolina at Chapel Hill, Chapel Hill, North Carolina, United States of America; 7 Injury Prevention Research Center, University of North Carolina at Chapel Hill, Chapel Hill, North Carolina, United States of America; University of Haifa, ISRAEL

## Abstract

**Background:**

Cancer treatment misinformation (e.g., false cures) is shared widely on social media and harmful. Cancer treatment misinformation is potentially shared because people want to help or provide hope for those with cancer. We need strategies, like prompts that inform others that a post may be false, to redirect individuals to prosocially intervene instead of sharing to reduce cancer treatment misinformation.

**Objective:**

We examined whether social cue prompts with a post review policy would lead to more intervening and less sharing of cancer treatment misinformation.

**Methods:**

We conducted a between-persons online experiment with adult participants from the US recruited via Prolific. Participants were randomized to view cancer treatment misinformation social media posts with social cue prompts and a flagging policy (treatment) or no prompts or policy (control) and reported willingness for intervening (e.g., flagging), sharing, and message reactions. Participants also reported their motivations for intervening or sharing the posts.

**Results:**

Social cue prompts and policies for platform action encouraged participants to intervene (e.g., flag) significantly more than people who did not see prompts, *p* < .001. Social cue prompts and policies also led to significantly less sharing and sharing actions on social media (e.g., liking posts), *p* < .001. Social cue prompts (vs. no prompts) led to greater motivation to intervene with the misinformation, most often because it could be false (accuracy concerns) and potentially harmful to people with cancer (empathic concerns), both *p* < .001. Social cue prompts also led to less motivation to share; fewer would share because the post was interesting or may provide hope (altruistic motivations), both *p* < .001.

**Conclusion:**

Prompts (also called warnings, nudges, or labels) on cancer treatment misinformation are a promising approach to encourage intervening (flagging) and reduce sharing on social media. Social cue prompts and policies also reduced common motivations for sharing – to help and provide (false) hope – that could be interpreted as misguided altruism.

## Introduction

Cancer misinformation that is false and potentially harmful is often shared on social media [1]. Specifically, cancer treatment misinformation on social media are posts about cancer treatments and cures that are not supported by current scientific consensus [2]. Cancer treatment misinformation can lead to psychosocial harm if individuals with cancer and cancer caregivers experience distress or abandon cancer support resources after exposure to harmful content [3]. Cancer treatment misinformation can also cause physical harm. Individuals with cancer have reduced survival if they follow misinformation advice for unproven therapies [4], or are likely to experience worse health outcomes if their caregivers are distressed [5].

Cancer misinformation represents upwards of 30–77% of cancer posts that are shared widely on social media [6–8]. Although there are some bad actors, cancer misinformation is also shared by friends and family of individuals with cancer and caregivers [9]. It is important to understand what motivations drive sharing cancer treatment misinformation to inform strategies to reduce sharing and potentially redirect efforts toward intervening to minimize harm [10].

People are motivated to share health and other (mis)information on social media for many reasons. Posts may simply be interesting, believable, or relevant for someone you know [11–14]. A host of theories point to plausible motivations for sharing. According to the preference-based account of (mis)information sharing, online sharing is driven by personal assessments (e.g., whether something is interesting or surprising) over accuracy determinations [11,13,14]. It is also possible that without interventions to debunk (or pre-bunk) cancer treatment misinformation, people are more likely to believe cancer treatment misinformation when they see it online [12]. According to the value-based theory of sharing, people also share information online to fulfill their motivations to be socially relevant [15,16]. Thus, people may share misinformation because they are motivated to support and help loved ones, especially those with cancer [17–21]. Sharing on social media may also be driven by altruistic motivations known to guide other cancer support behaviors (e.g., encouraging clinical trial enrollment) [22]. However, altruistic motivations for sharing cancer misinformation are misguided, despite good intentions [23].

To reduce cancer treatment misinformation on social media, we need evidence-based strategies. We relied on human-computer interaction (HCI) adaptions of the Bystander Intervention Model [24–28] and Social Presence Theory [29] to design an intervention to encourage intervening by flagging or reporting misinformation [30]. Flagging and reporting are small, albeit powerful, actions that can be performed by many to greatly reduce exposure to cancer treatment misinformation. There are five critical steps to effectively encourage prosocial intervening on social media, according to HCI adaptions of the Bystander Intervention Model [24–28]. To intervene, individuals must 1) notice the misinformation (attention), 2) perceive the severity for vulnerable individuals (empathy), 3) believe their actions are critical (responsibility), and 4) know how to intervene to be able to 5) act. With cancer treatment misinformation on social media, this process can be encouraged with prompts (also called nudges, warnings, or labels) on posts coupled with clear policies for action (e.g., if flagged by a certain number of people, the post will be removed for review). Specifically for misinformation, prompts may be a critical intervention to shift attention (noticing) for accuracy assessments that precede online bystander intervening, as well as to improve the quality of online sharing according to the inattention-based account for sharing misinformation [11].

Prompts that a social media post is false or potentially harmful reduce sharing and misperceptions about health information [31–33]. Ample evidence shows that simple prompts alerting viewers to inaccuracies or disputed information [31–33], or prompts that merely remind viewers to think about (in)accuracies [11,34–36], reduce sharing. Emerging evidence suggests prompts are more effective for reducing sharing when enhanced with social cues that communicate the online (social) presence and actions of others [30,37–40]. Social cues can encourage online prosocial behavior by signaling useful social information (i.e., descriptive norms) [41,42] – seeing that a few people (e.g., 8) or many people (e.g., 48) have already flagged a post as false and potentially harmful signals the actions and thoughts of others. Although prompts can be enhanced with social cues, there is some evidence that weak social cues (e.g., low dose with few people) may not have the same benefits as stronger doses (e.g., many people) for encouraging prosocial behaviors [41,42].

Prompts with social cues (social cue prompts) will likely motivate intervening through emotional and cognitive mechanisms supported by Social Presence Theory and the Bystander Intervention Model [26,27,29,30]. Social cues can increase the social presence or feeling of others in digital communication [29]. Seeing others’ actions in social cues increases the usefulness of online health information and can motivate action [43]. People may be motivated to intervene with social cue prompts because of accuracy concerns (noticing misinformation, step 1), out of empathic concern for vulnerable individuals with cancer (step 2), or feel responsibility to act (step 3). Information about how many other people already intervened (e.g., flagged) also sets up potential “tipping points” for increased responsibility. If paired with clear post-removal policies (e.g., remove post for review if 10 or 50 people flag), viewers can assess how influential their actions will be – a critical step in the Bystander Intervention Model. Prompts may also encourage action through aversive reactions (more negative affect, less positive affect) [44]. If people are sharing misinformation to help, redirecting these efforts to intervene with cancer treatment misinformation instead of sharing is a promising approach.

To assess the impact of social cue prompts and flagging polices on intervening and sharing cancer treatment misinformation, we conducted a between-persons online experiment. Participants were randomized to view cancer treatment misinformation posts with social cue prompts and a flagging policy (treatment) or no prompts and policy (control). We hypothesized that prompts and policies (vs. none) would encourage intervening (H1), reduce sharing (H2), and lead to more negative affect (and less positive affect) when viewing the posts (H3). Among those who saw the prompts and policy (treatment only), we examined whether showing a high dose (many people) or low dose (few people) of the social cues in the prompts and policies would impact flagging (RQ1). We also examined motivations for intervening with or sharing cancer treatment misinformation, whether these motivations differed when social cue prompts and policies were shown or not (RQ2), and details for whom and how they may share the misinformation in social media posts (RQ3). Last, we explored whether intervening, sharing, or motivations for intervening or sharing differed among those who are in cancer care networks (close to someone with cancer, cancer caregiver, or have cancer) and those who are not (RQ4).

## Methods

### Participants

From May 2 to May 4, 2024, we recruited a convenience sample of adult participants (*N* = 1051) using Prolific, an online survey research platform that maintains a panel of over 150,000 active users. Participants on Prolific are highly engaged and provide meaningful, high-quality responses [45,46]. Participants were eligible if they were at least 18 years old and a resident of the United States. The survey was only available online in English. Participants who completed the survey received $2.75 (the equivalent of an hourly wage of $16.50). All study procedures were reviewed and designated as exempt by the University of North Carolina Institutional Review Board 24–0460. Our reporting is guided by the American Psychological Association’s Journal Article Reporting Standards for Quantitative Research Designs (JARS-Quant) [47].

### Procedure

Individuals clicked the link to the Qualtrics survey in the Prolific platform and consented to participate with an online written consent form that stated they have read the information, voluntarily agree to participate, and are over 18 years of age before beginning the study. Following consent, participants shared basic demographics (i.e., age, race and ethnicity, gender) and political affiliation. After exposure to two fictional political advertisements for a separate study reported elsewhere [48], participants completed the cancer treatment misinformation experiment portion of the survey. Participants were randomized to see either misinformation posts with prompts and a policy for intervening (treatment) or no prompts or policy (control). Within the treatment conditions, participants were shown either a high dose (more people in the social cue) or low dose (fewer people) version of the prompts and policy (see Stimuli below). Simple randomization was done with Qualtrics’ built-in randomizer tool, which uses a pseudo-random number generator (PRNG), to allocate participants evenly (50/50) between the treatment and control conditions [49]. The algorithm randomly assigned embedded data for condition assignment for each respondent. For the treatment, participants were randomly assigned to one of two embedded data conditions—high dose or low dose—defined by distinct embedded data values. For the control condition, participants were randomly assigned to one of two equivalent embedded data options with identical values. This procedure ensured that each participant had an equal probability of being assigned to any of the four embedded data options, without stratification or blocking. Participants were blind to their condition allocation, and the research team was unaware of individual allocations until data collection was complete.

Participants were introduced to our fictional social media platform, branded as *Invibe*. Participants were told they were reviewing posts from a “beta version” of a new social media platform. We also stated that although *Invibe* “will allow users to swipe between posts, like (heart), comment, flag, dislike, and mute other users,” participants would, “simply view each post and its caption” in this study. On the following webpage of the survey, participants saw a static image of the *Invibe* welcome page with the flagging policy (treatment) or the platform branding (control). Participants then viewed two cancer treatment misinformation posts, presented in a random order. The misinformation posts were shown at the top of the webpage with all items about the post shown below. Last, participants shared more about their social media use, cancer experiences (close to someone with cancer, cared for someone with cancer, have been diagnosed with cancer, or none of these), and additional demographic information. If participants reported being close to someone with cancer or having cared for someone with cancer, they were asked a follow-up question about the relationship to the person with cancer. Participants with a previous cancer diagnosis were asked additional questions about the type of cancer, stage at diagnosis, and current treatment status.

### Stimuli

**Fig 1:**
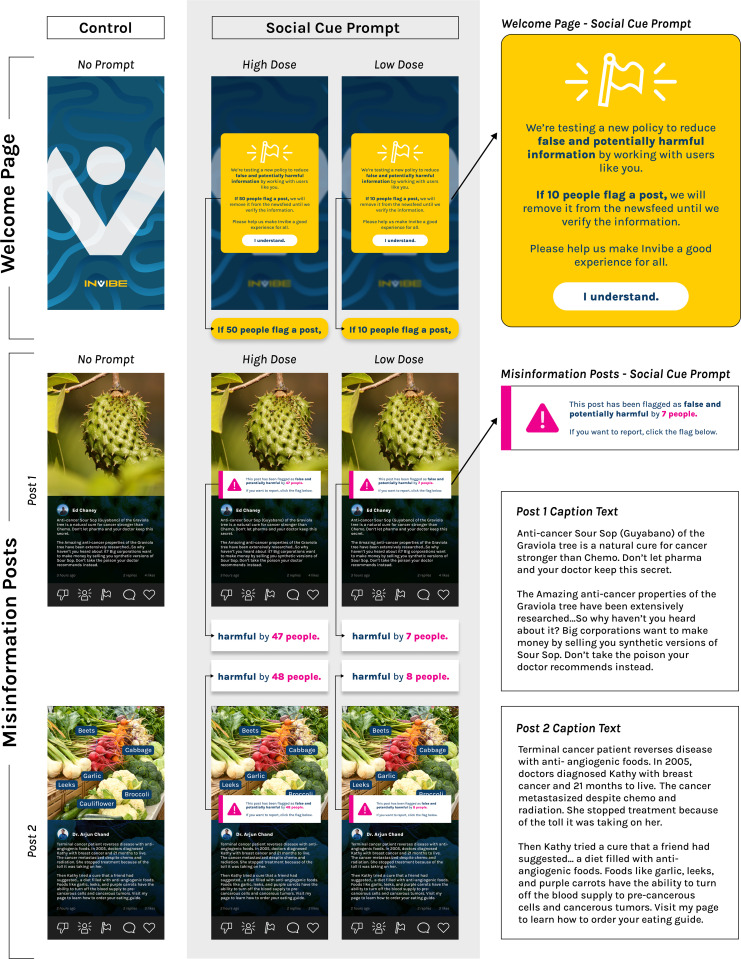
Stimuli.

Participants saw two cancer treatment misinformation posts adapted from our previous research (Fig 1) [30]. Cancer treatment misinformation is defined as information about treatments or cures that is false based on current scientific consensus [2]. One post was about soursop (guyabano) as a natural cure for cancer that is stronger than chemo but kept secret by big corporations making money and your doctor who recommends poison. The other post was about how anti-angiogenic vegetables (e.g., garlic, leeks) were able to cure a woman with metastasized breast cancer when chemo and radiation were unsuccessful. Both posts were created to mirror the style and content of actual posts found on Instagram by the research team, are commonly shared cancer treatment misinformation themes (e.g., natural cures) [6,50,51], and use persuasive strategies common to health misinformation (e.g., personal narratives, distrusting the government or pharmaceutical companies) [52]. The content for each post was vetted by two oncologists to verify that the advice did not follow current scientific consensus for clinical care and did not contain a promising future treatment (e.g., no viable mechanism for treatment, not currently in clinical trials).

Participants in the social cues (treatment) condition saw social cue prompts and a policy. The prompts to encourage intervening were shown on the post image, just above the caption, to increase likelihood of visibility [33]. We had two versions of the prompts and policies to explore whether the “dose” or number of people in the social cues made an impact on post reactions. Those with the high dose (more people) social cues saw the *Invibe* policy on the welcome page that “If 50 people flag a post, we will remove it from the newsfeed until we verify the information” before viewing the two stimuli posts. In the high dose social cue condition, the prompt on the post stated, “This post has been flagged as false and potentially harmful by [47 or 48] people. If you want to report, click the flag below.” The low dose (fewer people) social cue policy stated that if 10 people flagged then the post would be taken down for review, and the prompts stated that 7 or 8 people had flagged the post already.

### Measures

#### Intervening.

We assessed participants’ willingness to intervene with adapted items for three different actions [50]. For flagging (primary outcome), we asked, “How willing would you be to flag this post (for other users to see the flag)?” We asked similar items for our secondary outcomes of disliking, “How willing would you be to dislike (thumbs down) this post,” and muting, “How willing would you be to mute the account (to not see posts from this account in the future)?” Responses were “not at all willing” (coded as 1), “slightly willing” [2], “somewhat willing” [3], “moderately willing” [4], and “extremely willing” [5]. Intervening outcomes had high inter-item correlations indicating strong internal consistency (*r* = .72−.73). We averaged intervening outcomes across the two posts.

#### Sharing.

Willingness to share on social media was assessed with two adapted items [50] for liking, “How willing would you be to like (thumbs up) this post” and for commenting, “How willing would you be to comment on this post?” Responses ranged from “not at all willing” (coded as 1) to “extremely willing” [5] and were averaged across the posts given their high inter-item correlations indicating internal consistency (both *r* = .70). Given the possibility that participants would comment to refute misinformation (both sharing and intervening), we asked a clarifying question about comment valence, “If you were to comment, what type of comment would you post?” Response options were “comment to agree,” “comment to disagree,” “other,” or “I would not comment.” We recoded the response options to create three new dichotomous variables: comment to agree, comment to disagree, and other. If respondents indicated they would comment to agree with either post, they received a 1 response for commenting to agree. If they did not indicate they would comment to agree with either post, they received a 0 for that variable.

We assessed sharing, in general, with the item [53], “How likely are you to share this post with others?” Response options ranged from “not at all likely” (coded as 1) to “extremely likely” [5] and were averaged across the two posts given their high inter-item correlations indicating internal consistency (*r* = .70). Participants then reported how they would share by selecting all channels they would use or that they would not share in any of these ways (mutually exclusive) [50,53]. Participants reported if they would share the post with “someone with cancer,” “someone who is caring for someone with cancer,” or “someone who does not have cancer” across three items. Responses were select all that apply for family (immediate or other), friends, acquaintances (someone I know), other, or that they would not share (mutually exclusive) [53]. Each response option was dichotomized; where participants indicated an affirmative response to either item across the two posts, they were coded as 1.

#### Affective reactions.

We also assessed affective reactions to better understand how the posts made participants feel. We modified an item to capture positive affect and negative affect uniquely: “How [good/bad] does viewing this post make you feel?” Response options ranged from “not at all” (coded as 1) to “extremely” [5] and were averaged across the two posts given their high inter-item correlations indicating internal consistency (*r* = .73−.75).

#### Motivations for intervening and sharing.

To assess motivations or reasons for intervening and sharing, we created two “select all that apply” exploratory items. We asked, “why would you intervene with (e.g., flag, report) this post?” with six motivations and the option that they would not intervene (mutually exclusive). Motivations for intervening were adapted or created to capture the cognitive and emotional predictors of intervening (e.g., empathy, perceived responsibility) from HCI adapted versions of the Bystander Intervention Model and from the inattention-based account for misinformation sharing across many topics [11,26,54,55]. Similarly, we asked, “why would you share this post?” with nine motivations and an option that they would not share (mutually exclusive). Motivations for sharing (e.g., sharing interesting, helpful, relevant information) were adapted or created from studies for (mis)information sharing on social media and cancer support based on theory and evidence from the preference-based account of misinformation sharing, the value-based theory of sharing, the value-based virality model, and inoculation theory [11–15,17,18]. Each response option was dichotomized; where participants indicated an affirmative response to either item across the two posts, they were coded as 1.

### Data analyses

Prior to analyses, we removed responses from participants who completed the survey implausibly fast (less than 60 seconds, *n* = 8). Then we confirmed that participants were randomized to the social cue prompt and policy (high dose/low dose) and the stimuli without prompts as intended. Next, we compared the two social cue conditions (high dose/low dose) on flagging to assess the differential impact and to determine if we would analyze the social cue prompts (treatment) as collapsed or separately across all outcomes. We estimated general linear models for the intervening, sharing, and affective reaction outcomes. Each model included a 2-level categorical fixed effect for condition (social cue prompt and policy vs. no-prompt control). We also explored whether being in a cancer care network (close to someone with cancer, cancer caregiver, or had cancer) vs. not moderated the impact of the prompts for intervening. Chi-square tests were used to examine respondents’ motivations for intervening and sharing. We provide the general linear model results by individual post for intervening, sharing, and affective reaction outcomes (Appendix A) and the correlograms with phi coefficients for the binary motivation outcomes for intervening and sharing in the appendices (Appendix B-D). All models used individual responses as the unit of analysis. The data are available at https://doi.org/10.17615/knd1-fw1.

## Results

Adult participants (*N* = 1051) were, on average, 43 years old (*SD* = 13.17, Table 1). Participants were half women (50%) and identified mainly as non-Hispanic white (63%) or as Black or African American (15%). Most participants used social media daily (91%). More than one in three participants were close to someone with cancer (38%), including immediate family members (*n* = 190), another family member (*n* = 57), and friends (*n* = 44); and one in four were cancer caregivers (25%) for immediate family members (*n* = 239), another family member (*n* = 145), and friends (*n* = 118). Few participants had a cancer diagnosis (5%), including breast (*n* = 14), Melanoma (*n* = 5), non-Melanoma skin (*n* = 5), testicular (*n* = 5), and thyroid (*n* = 5) cancers, among other. Most reported stage I (*n* = 21) or stage II (*n* = 15) diagnoses and had completed treatment (*n* = 36).

**Table 1:** Participant characteristics (*N* = 1051).

	Overall	No prompt control, *n* = 501	Social cue prompt, *n* = 550	Condition comparisons	
	*M (SD)*	*M (SD)*	*M (SD)*	*t*	*p*
Age, *M (SD)*	42.51 (13.17)	43.42 (13.30)	41.69 (13.01)	2.13	.03
	*n* (%)	*n* (%)	*n* (%)	*χ* ^*2*^	*p*
Race or ethnicity				3.09	.96
Non-Hispanic white	661 (63%)	318 (63%)	343 (62%)		
Hispanic, Latino, or Spanish	57 (5%)	29 (6%)	28 (5%)		
Black or African American	161 (15%)	77 (15%)	84 (15%)		
Asian	66 (6%)	34 (7%)	32 (6%)		
Another race or ethnicity	10 (1%)	4 (1%)	6 (1%)		
Multi-racial	91 (9%)	37 (7%)	54 (10%)		
Gender				.35	.84
Woman	527 (50%)	267 (53%)	260 (47%)		
Man	511 (49%)	229 (46%)	282 (51%)		
Non-binary	11 (1%)	4 (0.8%)	7 (1%)		
Transgender				1.71	.64
Yes, transgender	22 (2%)	11 (2%)	11 (2%)		
No, not transgender	1023 (97%)	487 (97%)	536 (97%)		
Cancer experience					
Previous cancer diagnosis**	50 (5%)	30 (6%)	20 (4%)	3.20	.07
Cancer caregiver***	261 (25%)	132 (26%)	129 (23%)	1.18	.28
Close to someone with cancer****	405 (38%)	191 (38%)	213 (39%)	.04	.84
Social media use*				1.34	.72
Daily	956 (91%)	456 (91%)	500 (91%)		
Weekly	54 (5%)	28 (6%)	26 (5%)		
Less than weekly	23 (2%)	9 (2%)	14 (3%)		
Do not use social media	17 (2%)	7 (1%)	10 (2%)		
Political Affiliation				1.71	.63
Republican	515 (49%)	250 (50%)	265 (48%)		
Democrat	510 (48%)	236 (47%)	274 (50%)		
Independent	23 (2%)	13 (3%)	10 (2%)		

*Participants reported using YouTube (*n* = 842, 81%), Facebook (*n* = 799, 77%), Instagram (*n* = 705, 68%), Reddit (*n* = 663, 64%), Twitter/X (*n* = 549, 53%), TikTok (*n* = 473, 46%), Snapchat (*n* = 294, 28%), Truth Social (*n* = 26, 2%), or other social media (*n* = 31, 3%).

**Participants who reported a previous cancer diagnosis identified the type of cancer, stage at diagnosis, and current treatment status.

Cancer: breast (*n* = 14, 28%), Melanoma (*n* = 5, 10%), non-Melanoma skin (*n* = 5, 10%), testicular (*n* = 5, 10%), thyroid (*n* = 5, 10%), gynecologic (*n* = 4, 8%), bladder (*n* = 3, 6%), colorectal (*n* = 3, 6%), or other.

Stage: I (*n* = 21, 42%), II (*n* = 15, 30%), III (*n* = 6, 12%), IV (*n =* 1, 2%), or not applicable (*n* = 7, 14%).

Treatment status: haven’t started treatment (*n* = 2, 4%), currently receiving treatment (*n* = 6, 12%), between treatments (*n* = 5, 10%), completed treatment (*n* = 36, 72%), or in hospice care (*n* = 1, 2%).

***Participants reported being cancer caregivers for immediate family member (*n* = 190, 73%), another family (*n* = 57, 22%), friend (*n* = 44, 17%), or other (*n* = 12, 5%).

****Participants were close to someone with cancer who is an immediate family member (*n* = 239, 59%), another family member (*n* = 145, 36%), friend (*n* = 118, 22%), or other (*n* = 4, 1%).

For all analyses, we collapsed the two social cue conditions (high dose/low dose) after comparing the means on the primary outcome of flagging; the social cue prompts did not significantly differ, *p* = .20, for willingness to intervene by flagging (high dose: *M* = 3.02, *SD* = 1.51; low dose: *M* = 2.85, *SD* = 1.52). Dose did not impact intervening for RQ1. Participants in the treatment (social cue prompts and policy) and control conditions did not differ on race or ethnicity, gender, identifying as transgender, cancer experience, social media use, or political affiliation. There were condition differences for age at baseline. The average age in the control condition was 43 years old (SD = 13.30), while the average age in the social cue condition was 42 years old (SD = 13.01). In a sensitivity analysis, adding age as a covariate did not impact the significance or direction of the condition effects (social cue vs. control) on our primary outcome (willingness to flag), thus we report unadjusted analyses below.

### Prompts encouraged intervening and discouraged sharing

Social cue prompts, along with their policy for platform action, encouraged intervening. Participants who saw the cancer treatment misinformation posts with social cue prompts were significantly more willing to flag a post than those who did not see the prompts, *F*(1,1048) = 111.6, *p* < .001, Table 2. H1 was supported. The social cue prompts led people to be, on average, “somewhat willing” to flag posts, while those in the no prompt control were “slightly willing” to flag – a medium-to-large effect (Appendix E). Similarly, social cue prompts (vs. no prompt) led to greater willingness to dislike cancer treatment misinformation posts, *F*(1,1048) = 62.86, *p* < .001, and willingness to mute those who shared the misinformation, *F*(1,1048) = 49.38, *p* < .001 – both medium-sized effects encouraging people to be “somewhat” or “moderately” willing to intervene (vs. “slightly” willing). Intervening actions were similar among those in cancer care networks (close to someone with cancer, cancer caregiver, or had cancer) and those who were not. All interactions for flagging, *F*(1,1045) =.00, *p* = .944, disliking, *F*(1,1045) =.52, *p* = .470, and muting, *F*(1,1045) = 91, *p* = .340, were not significant.

**Table 2:** Intervening, sharing, and affective reactions to cancer misinformation posts.

	No prompt *n = 501*	Social cue prompt *n* = 550			Cohen’s *d (*95% CI)
	*M (SD)*	*M (SD)*	*F*	*p*	
**Intervening**					
Flag	2.00 (1.312)	2.93 (1.52)	111.6	<.001	.66 (.53,.78)
Dislike	1.97 (1.37)	2.67 (1.50)	62.86	<.001	.49 (.36,.61)
Mute	2.52 (1.54)	3.18 (1.50)	49.38	<.001	.43 (.31,.56)
**Sharing**					
Like	2.00 (1.28)	1.55 (1.00)	40.45	<.001	.39 (.27,.52)
Comment	1.88 (1.20)	1.76 (1.11)	2.63	.11	.10 (−.02,.23)
Agree, *n* (%)	128 (26%)	72 (13%)	--	--	
Disagree, *n* (%)	89 (18%)	156 (28%)	--	--	
Other, *n* (%)	29 (6%)	21 (4%)	--	--	
Share (general)	1.94 (1.22)	1.53 (0.94)	37.68	<.001	.38 (.26,.50)
**Affective reactions**					
Positive affect	2.23 (1.28)	1.66 (0.99)	66.23	<.001	.50 (.38,.62)
Negative affect	2.07 (1.30)	2.55 (1.32)	35.10	<.001	.37 (.24,.49)

*Note:* Responses included the value labels of “not at all” (coded as 1), “slightly” (2), “somewhat” (3), “moderately” (4), and “extremely” (5).

Social cue prompts also led to lower willingness to share posts – through social media actions and in general (various channels) – both small-to-medium effects. Participants who saw the social cue prompts and policy were significantly less willing to like a post than those without prompts, *F*(1,1049) = 40.45, *p* < .001. Participants were also less likely to share about the cancer misinformation when they saw the posts with social cue prompts (vs. without prompts), *F*(1,1049) = 37.68, *p* < .001. H2 was supported. Few people were willing to comment whether there were prompts or not, but the valence of the comments varied between the conditions. Fewer people would comment to agree in the social cue prompts condition (13%) than in the no prompts control condition (26%). More people would comment to disagree in the social cue prompts condition (28%) than in the no prompts control condition (18%). Sharing (by liking a post or in general) was similar among people in cancer care networks and those who were not. Interactions were not significant for liking, *F*(1,1046) = 1.04, *p* = .308, or sharing about the cancer misinformation *F*(1,1046) = 2.47, *p* = .116.

Participants reported they would share about misinformation posts across a variety of channels and with people impacted by cancer, albeit less so if the post had prompts (Table 3). Half as many participants would share via any channel when posts had social cue prompts (17%) versus no prompts (32%). The pattern of social media, text messaging, and in-person channels used to share were similar across conditions with one exception, addressing RQ3. Social cue prompts had the greatest impact for less sharing on social media profiles where posts can be viewed publicly on feeds; 13% would share on a profile with prompts shown versus 25% with no prompts. The participants who would share reported they would be similarly willing to share cancer misinformation posts with individuals with cancer, caregivers, and people without cancer when prompts are shown (11–18%) or not (19–31%).

**Table 3:** Cancer misinformation sharing channels and recipients.

	No prompt *n = 501*	Social cue prompt *n* = 550
	*n (%)*	*n (%)*
**Would share via...**		
Repost on social media profile	123 (25%)	74 (13%)
Repost on social media story (temporary)	76 (15%)	53 (10%)
Direct (private) message on social media	80 (16%)	62 (11%)
Text message	83 (16%)	53 (10%)
In-person	77 (15%)	53 (10%)
Email	38 (7%)	20 (4%)
Would not share	339 (68%)	456 (83%)
**Would share with…**		
Someone with cancer		
Immediate family member with cancer	154 (31%)	81 (15%)
Friend with cancer	135 (27%)	80 (15%)
Other family member with cancer	132 (26%)	66 (12%)
Someone I know with cancer	131 (26%)	73 (13%)
Would not share with someone with cancer	358 (74%)	472 (86%)
Someone who is a cancer caregiver		
Immediate family member cancer caregiver	155 (31%)	85 (15%)
Friend cancer caregiver	142 (28%)	84 (15%)
Other family member cancer caregiver	130 (26%)	69 (13%)
Someone I know cancer caregiver	125 (25%)	66 (12%)
Would not share with a cancer caregiver	354 (71%)	467 (85%)
Someone who does not have cancer		
Immediate family member	114 (23%)	82 (15%)
Friend	114 (23%)	97 (18%)
Other family member	96 (19%)	60 (11%)
Someone I know	102 (20%)	58 (11%)
Would not share with someone without cancer	381 (76%)	469 (85%)

*Note:* Items were “select all that apply” so percentages may exceed 100%.

Viewing the social cue prompts also impacted how people felt when viewing the posts. People felt significantly less positive when viewing the cancer treatment misinformation with the social cue prompts (vs. no prompts), *F*(1,1049) = 66.23, *p* < .001. Conversely, individuals felt significantly worse when viewing the misinformation posts with the social cue prompts (vs. no prompts), *F*(1,1049) = 35.10, *p* < .001. H3 was supported.

### Prompts led to greater motivations to intervene

Participants were motivated to intervene with the misinformation because of it being false and potentially harmful (Table 4). Notably for RQ2, with the social cue prompts (vs. no prompt), significantly more people were motivated to intervene because the post “could be false” (accuracy concern), they were “concerned about people with cancer who may be harmed” (empathic concern), and “others may be susceptible” (empathic concern), all *p* < .001. These motivations were also endorsed by the highest number of people: “could be false” (47–61%), concerned about those “who may be harmed” (33–44%), and “others may be susceptible” (27–36%). Although perceived responsibility motivations (e.g., “feel personally responsible,” “would feel guilty if didn’t”) were endorsed by almost one in five participants (17–22%), these motivations were endorsed by the fewest people and did not differ with or without the prompts. While all motivations for intervening were fairly to moderately correlated (Appendix B-C), concerns about harms and susceptibility, ϕ = .54, *p* < .001, and feeling responsible and guilty, ϕ = .54, *p* < .001, had the strongest correlations.

**Table 4:** Motivations for sharing and intervening with cancer misinformation posts.

	No prompt *n = 501*	Social cue prompt *n* = 550		
	*n (%)*	*n (%)*	*X* ^*2*^	*p*
**Intervening motivations**				
This information could be false.	238 (47%)	337 (61%)	20.06	<.001
I am concerned about people with cancer who may be harmed.	168 (33%)	244 (44%)	12.90	<.001
Others may be susceptible to this information.	136 (27%)	197 (36%)	9.11	<.001
I feel personally responsible to intervene so no one is harmed.	99 (20%)	122 (22%)	0.92	.34
I would feel guilty if I didn’t intervene.	84 (17%)	112 (20%)	2.24	.13
Other	10 (2%)	9 (2%)	0.19	.66
Would not intervene	276 (55%)	230 (42%)	18.50	<.001
**Sharing motivations**				
This information is interesting.	205 (41%)	124 (22%)	41.15	<.001
This information may provide hope for someone with cancer.	137 (27%)	82 (15%)	24.58	<.001
This information could help someone whose cancer treatment isn’t working	120 (24%)	70 (13%)	22.30	<.001
People should know about this cancer information.	119 (24%)	63 (11%)	27.69	<.001
This post has a promising cure for cancer.	99 (20%)	54 (10%)	20.83	<.001
This information is believable.	97 (19%)	69 (12%)	9.16	<.01
This information is relevant for someone I know.	90 (18%)	55 (10%)	13.98	<.001
This information is surprising.	90 (17%)	67 (12%)	6.90	<.01
Other	18 (3%)	11 (2%)	2.48	.11
Would not share	321 (64%)	436 (79%)	30.07	<.001

Note: If someone endorsed a reason for either of the two posts, they are included in this count (0 = not a reason, 1–2 = is a reason/motivation).

### Prompts led to fewer motivations to share

People were motivated to share cancer treatment misinformation for a variety of reasons. For all sharing motivations addressed in RQ2, almost twice as many participants endorsed the motivation for sharing when there were no prompts vs. social cue prompts shown (Table 4). Without prompts, the most endorsed motivations to share were that the misinformation posts were “interesting” (41%), “may provide hope for someone with cancer” (27%), “could help someone whose cancer treatment isn’t working” (24%), and that “people should know about the cancer information” (24%). All these motivations were endorsed by significantly fewer people when the social cue prompts are shown on the posts. With a social cue prompt, fewer people were motivated to share because the misinformation post was “interesting” (22%), “may provide hope” (15%), “could help” if treatment was not working (13%), or that “people should know” (11%). Sharing motivations were all fairly to strongly correlated (Appendix D), with the strongest correlations for providing hope and helping when treatment is not working, ϕ = .71, *p* < .001, and providing hope and people should know about this cancer information, ϕ = .63, *p* < .001.

## Discussion

Cancer treatment misinformation spreads widely on social media. We need effective strategies to encourage intervening to reduce the potential harm of this misinformation. Using prompts (also called nudges, warnings, or labels) on social media posts encourages intervening (e.g., flagging to report) with cancer misinformation. Our study supports and extends the evidence for effective strategies to encourage intervening to reduce cancer treatment misinformation, especially those that leverage showing others’ actions to motivate behavior [30,37–39,42].

We found social cue prompts (stating how many other people have flagged as false and potentially harmful) paired with platform policies to remove flagged posts effectively encouraged willingness to intervene. People were somewhat likely to intervene with the prompts and policies, a shift that represents a medium-sized effect. Even modest shifts in individuals’ willingness to intervene—such as being somewhat versus slightly willing to flag a post—can collectively generate meaningful impact if scaled among the many US adults who use social media for cancer support and sharing. These social cue prompts and policies also had desirable spillover effects; social cue prompts led to lower sharing intentions and more negative (affective) message reactions when viewing the cancer misinformation social media posts. Social cue prompts and policies also influenced motivations in desired ways. Fewer people had motivations for sharing and more people had reasons for intervening when misinformation posts had social cue prompts shown. Notably, our findings were consistent among individuals in cancer networks (with cancer, cancer caregivers, or close to someone with cancer) and the general population.

Showing social cue prompts paired with clear platform review policies to remove posts after flagged by enough people led to greater willingness for the targeted intervening behavior (flagging), as well as for other non-targeted intervening actions (muting, disliking). Giving participants straightforward direction for action inspired an openness to take directed action – as predicted in the HCI adapted Bystander Intervention Model. It is encouraging that specific “flag to report” instructions led to willingness for other (non-instructed) intervening behaviors as well. While the prompts and policy led to more willingness to intervene, it is possible that some participants did not fully understand the policy or believe and trust the prompts, which would have tempered the impact of the intervention. We should explore how comprehension, believability, and trust influence reactions to cancer misinformation interventions.

Having a variety of intervening options highlighted in prompts and policies would allow people to do what they feel most comfortable with (e.g., public vs. private actions, those with removal consequences vs. not). Options for prompts and intervening actions will likely increase as our social media landscape reshapes from legal challenges to exclusive platform control of algorithm-based feeds [56] and technical solutions for people to filter out problematic content (e.g., Perspective API uses machine learning to identify and reduce exposure to toxic content as defined by the user) [57]. Individuals and cancer support organization would benefit from customizable tools that allow them to identify and apply prompts to filter out cancer treatment misinformation or other harmful or distressing content (e.g., inordinate discussion of rare events, unwanted advice) [3,50]. As legacy platforms abandon their human-agent content moderation and use of micro social media for cancer support increases, our social media prompts and policies provide a set of features consistent with the needs of new platform administrators to minimize harmful content. Instead of burdening individuals to rely on inadequate “devised strategies” for managing harms of constant exposure, prompts and policies can be built into modifiable machine-learning or AI-based tools to filter harmful content.

We found that social cue prompts and policies with higher or lower doses (strengths) similarly encouraged intervening. While others have found that signaling many people (125 or more have flagged) versus few (5–25 people have flagged) in social cues or social norms for misinformation leads to greater likelihood of reporting posts [42], our evidence suggests that policies could be sensitive (only need 10 people to flag to remove for review) or more conservative (need 50 people to flag) to have the intended impact. It is also possible that our manipulation of few (needing 10 people to flag to review a post) versus many (50 people to flag for review) was not interpreted as low or high doses of these social cues. Future studies should continue to explore perceptions of social cues and thresholds for their effectiveness for motivating online behavior.

Overall, the social cue prompts also changed how people felt when viewing the posts. Participants felt less positive and more negative after viewing posts when the prompts stated the post had been previously flagged as false and potentially harmful. Influencing affective reactions is a potentially strong mechanism for encouraging intervening action, given that it is well established that our affective responses often drive our quick or in-the-moment behaviors [44].

Participants most often reported being motivated to intervene because they had concerns about the accuracy of the (mis)information or empathy for those who may be negatively impacted by the posts. These motivations – accuracy and empathic concerns – were endorsed by more people when prompts were shown on the cancer treatment misinformation posts. Empathic concern has been shown to drive cyberbullying intervening and may be an important mechanism to encouraging intervening online broadly [27]. People were less likely to report that they would intervene because they felt responsible or guilty (with or without prompts), despite perceived responsibility being a mechanism for encouraging intervening in other circumstances [26]. Consistent with the human-computer interaction (HCI) adaptation of the Bystander Intervention Model, the strongest correlations were between items corresponding to the established constructs of empathy and perceived responsibility [24–28]. To our knowledge, this is the first study to explore motivations for intervening with a multi-response item that simplifies the assessment of a complex topic. We hope others will use this preliminary evidence as a foundation for rigorous psychometric validation and scale development and to advance the measurement of motivations underlying intervening behaviors in the context of cancer treatment and other health misinformation.

Social cue prompts and policies reduced willingness to share cancer treatment misinformation – a desirable spillover effect. People were, on average, “slightly” willing to share cancer treatment misinformation without prompts and would share less when prompts were shown. Overall, people who would share reported wanting to do so through social media (reposting to profiles, stories, or direct messages), texting, or in person. Notably, we saw the largest reduction in willingness to share through social media profile reposting (i.e., on public feeds where these posts could have the largest reach) when prompts were shown on the misinformation posts.

If people are willing to share, they report being motivated to share cancer misinformation because it is interesting or to be helpful. Interest in the post being the most endorsed motivation for sharing provides further evidence for the preference-based account of misinformation sharing, where people choose to share (mis)information based on their preferences rather than a focus on accuracy assessments [11]. The following most endorsed sharing motivations are in line with the value-based theory of sharing, with the most robust associations among items representing motivations to be socially relevant to others [15]. In other words, after being simply interested, some participants’ motivations to share cancer misinformation could be interpreted as misguided altruism – if people are sharing to provide informational support or hope, especially for those whose cancer treatment is not working. Future studies are needed to validate these items and to better understand if these motivations are altruistic (versus self-serving) or serve a different need for the person sharing.

Our exploratory evidence suggests that although individuals are trying to help, their sharing may be potentially harmful. This paradox should not be taken lightly. Fortunately, we can reduce sharing of cancer misinformation that does not align with current scientific consensus with prompts and policies. But not all cancer treatment misinformation shared on social media is clearly false. Some information shared on social media may represent promising mechanisms or treatments currently in trials. Thus, we should use misinformation prompts and policies prudently, to ensure we are reducing potentially harmful misinformation and not simply putting a chilling effect on cancer support. Many turn to social media for non-clinical cancer advice – thoughts and experiences from those who get cancer because they had cancer [3,58]. Social media sharing can help individuals with cancer deal with the many physical and psychological issues during treatment and survivorship (e.g., tips for dealing with treatment side effects, support for parenting with cancer) [3]. Prompts should not deter individuals from sharing their experiences to support others, as this is one of the most sought out and valued uses of cancer support on social media.[3] We should not expect everyone who can offer valuable cancer support to be aware of current scientific consensus, although current scientific consensus could very reasonably be a platform’s threshold for when posts include prompts (or not).

This study is limited to the cancer treatment misinformation and type of prompts and policies presented in the stimuli. Our stimuli were developed to mimic posts available on Instagram for topics that are commonly reported as unwanted advice from those with cancer and had no correct information (i.e., no information supported by current scientific consensus, no viable mechanism for treatment). However, misinformation in cancer posts on social media are on a continuum (some truth to wholly false). Reactions to other cancer misinformation, especially posts that have elements of truth alongside unsupported information, shared by trusted sources, or that leverage engaging imagery and emojis, may differ. Other prompt types (e.g., rebuttals, expert source corrections) or strategies (e.g., media literacy interventions) may also effectively encourage intervening and should be examined in contrast to or combination with social cue prompts and policies. These prompts and policies or other intervention types may be more or less effective among populations with varying levels of media literacy skills, which should be considered in future studies. This study relies on self-report data for willingness to intervene and share social media posts. Previously, we observed a consistent pattern between self-reported willingness to intervene and share and observed behavior with cancer misinformation in our simulated social media [30]; however, self-reported willingness may not lead to behavior on existing social media and may be influenced by social desirability bias. The findings may also be biased if participants perceived our simulated *Invibe* to be unrealistic. Future studies focused on ecological validity are needed to determine the real-world impact of prompts and policies.

## Conclusion

Cancer treatment misinformation is shared widely on social media, paradoxically by many who are trying to be helpful. Some sharing motivations could be interpreted as misguided altruism, which could be redirected. We need to encourage individuals to prosocially intervene with cancer treatment misinformation to reduce harm. We found that prompts on posts with social cues (i.e., messages with how many people have already flagged a post as false and potentially harmful) and clear platform review policies (i.e., post removed after flagged by certain number of people) encourage intervening and reduce sharing of cancer treatment misinformation on social media.

## Supporting information

S1 Appendix Intervening, sharing, and affective reactions for each cancer misinformation post. (PDF)

S2 Appendix Theoretical support for intervention and sharing motivations. (PDF)

S3 Appendix Correlogram for intervening motivations. (PDF)

S4 Appendix Correlogram for sharing motivations. (PDF)

S5 Appendix Plots of effect sizes. (PDF)
