# Supplementary material for: Intervening and reducing sharing of false cancer treatments on social media: Online experiment
Source: PLoS One. 2026 Feb 25;21(2):e0341907. doi: 10.1371/journal.pone.0341907 (PMC12935207; doi:10.1371/journal.pone.0341907)
Supplement: S1 Appendix — (PDF) [file pone.0341907.s001.pdf]

Appendix A. Intervening, sharing, and affective reactions for each cancer misinformation post

	**Post 1 (sour sop)**				**Post 2 (anti-angiogenic foods)**			
	No prompt *n = 501*	Social cue prompt *n* = 550			No prompt *n = 501*	Social cue prompt *n* = 550		
	M (SD)	*M (SD)*	*F*	*p*	*M (SD)*	*M (SD)*	*F*	*p*
**Intervening**								
Flag	2.06 (1.44)	2.98 (1.65)	91.04	<.001	1.93 (1.40)	2.89 (1.62)	102.04	<.001
Dislike	2.08 (1.50)	2.75 (1.64)	46.94	<.001	1.85 (1.42)	2.58 (1.63)	58.55	<.001
Mute	2.63 (1.65)	3.23 (1.62)	35.58	<.001	2.40 (1.63)	3.14 (1.64)	52.83	<.001
**Sharing**								
Like	1.88 (1.33)	1.51 (1.06)	24.18	<.001	2.08 (1.42)	1.58 (1.12)	40.83	<.001
Comment	1.82 (1.26)	1.79 (1.23)	0.24	.625	1.92 (1.31)	1.74 (1.20)	5.33	.021
Agree, *n* (%)	90 (18%)	50 (9%)	--	--	113 (23%)	56 (10%)	--	--
Disagree, *n* (%)	74 (15%)	129 (23%)	--	--	59 (12%)	121 (22%)	--	--
Other, *n* (%)	16 (3%)	14 (3%)	--	--	20 (4%)	13 (2%)	--	--
Share (general)	1.86 (1.29)	1.53 (1.02)	20.73	<.001	2.00 (1.32)	1.52 (1.04)	43.15	<.001
**Affective reactions**								
Positive affect	2.03 (1.32)	1.59 (1.03)	36.73	<.001	2.41 (1.43)	1.73 (1.12)	75.59	<.001
Negative affect	2.19 (1.40)	2.59 (1.41)	21.17	<.001	1.97 (1.35)	2.51 (1.43)	39.44	<.001

Note: Responses included the value labels of “not at all” (coded as 1), “slightly” (2), “somewhat” (3), “moderately” (4), and “extremely” (5).
