# Supplementary material for: Intervening and reducing sharing of false cancer treatments on social media: Online experiment
Source: PLoS One. 2026 Feb 25;21(2):e0341907. doi: 10.1371/journal.pone.0341907 (PMC12935207; doi:10.1371/journal.pone.0341907)
Supplement: S2 Appendix — (PDF) [file pone.0341907.s002.pdf]

Appendix B. Theoretical Support for Intervention and Sharing Motivations

**Intervening motivations**	
**Item [ID]**	**Theoretical support**
This information could be false. [False]	According to the **inattention-based account for sharing misinformation**, shifting attention to perceived (in)accuracy can improve the quality of information people choose to share or potentially intervene with.
I am concerned about people with cancer who may be harmed. [Concerned]	According to the **human-computer interaction (HCI) adaptation of the bystander intervention model**, empathy and perceived responsibility are key cognitive and emotional predictors for whether someone will intervene online.
Others may be susceptible to this information. [Susceptible]	
I feel responsible to intervene so no one is harmed. [Responsible]	
I would feel guilty if I didn't intervene. [Guilty]	
**Sharing motivations**	
**Item [ID]**	**Theoretical support**
This information is interesting. [Interesting]	According to the **preference-based account of misinformation sharing**, sharing is driven more by preferences (e.g., whether something is interesting or surprising) than accuracy assessments.<br>Additionally, surprise (or novelty) can be a positive response that motivates sharing, according to the **theory of social sharing of emotion.**
This information is surprising. [Surprising]	
This information may provide hope for someone with cancer. [Hope]	According to the **value-based theory of sharing**, sharing support can help fulfill motivations to be socially relevant to others (e.g., connecting to provide hope, helping someone whose treatment is not working).
This information could help someone who's cancer treatment isn't working. [Treatment]	
People should know about this cancer information. [Know]	According to the **value-based virality model**, someone's choice to share online is influenced by the relative value of the information to oneself. Information can be relatively valuable for many reasons that vary (e.g., see a promising cure, identify information others should know, perceive information to be relevant).
This post has a promising cure for cancer. [Cure]	
This information is relevant for someone I know. [Relevant]	
This information is believable. [Believable]	Based on **inoculation theory**, people are less likely to believe a claim that has been debunked (refuted after exposure) or pre-bunked (refuted before exposure).
