# Supplementary material for: Intervening and reducing sharing of false cancer treatments on social media: Online experiment
Source: PLoS One. 2026 Feb 25;21(2):e0341907. doi: 10.1371/journal.pone.0341907 (PMC12935207; doi:10.1371/journal.pone.0341907)
Supplement: S3 Appendix — (PDF) [file pone.0341907.s003.pdf]

Appendix C. Correlogram for Intervening Motivations

**Item [ID]**	False	Concerned	Susceptible	Responsible	Guilty
This information could be false. [False]	1.00				
I am concerned about people with cancer who may be harmed. [Concerned]	0.50***	1.00			
Others may be susceptible to this information. [Susceptible]	0.50***	0.54***	1.00		
I feel personally responsible to intervene so no one is harmed. [Responsible]	0.30***	0.50***	0.39***	1.00	
I would feel guilty if I didn’t intervene. [Guilty]	0.23***	0.44***	0.41***	0.56***	1.00

Note: ***p < .001
