# Supplementary material for: Intervening and reducing sharing of false cancer treatments on social media: Online experiment
Source: PLoS One. 2026 Feb 25;21(2):e0341907. doi: 10.1371/journal.pone.0341907 (PMC12935207; doi:10.1371/journal.pone.0341907)
Supplement: S4 Appendix — (PDF) [file pone.0341907.s004.pdf]

Appendix D. Correlogram for Sharing Motivations

**Item [ID]**	Interesting	Hope	Treatment	Know	Cure	Believable	Relevant	Surprising
This information is interesting. [Interesting]	1.00							
This information may provide hope for someone with cancer. [Hope]	0.52***	1.00						
This information could help someone whose cancer treatment isn't working. [Treatment]	0.47***	0.71***	1.00					
People should know about this cancer information. [Know]	0.51***	0.63***	0.60***	1.00				
This post has a promising cure for cancer. [Cure]	0.41***	0.51***	0.49***	0.57***	1.00			
This information is believable. [Believable]	0.48***	0.40***	0.39***	0.48***	0.49***	1.00		
This information is relevant for someone I know. [Relevant]	0.44***	0.43***	0.45***	0.50***	0.50***	0.43***	1.00	
This information is surprising. [Surprising]	0.46***	0.36***	0.34***	0.39***	0.41***	0.38***	0.32***	1.00

Note: ***$p < .001$
