# Supplementary figures and images for: Intervening and reducing sharing of false cancer treatments on social media: Online experiment
Source: PLoS One. 2026 Feb 25;21(2):e0341907. doi: 10.1371/journal.pone.0341907 (PMC12935207; doi:10.1371/journal.pone.0341907)

## Appendix E. Plots of Effect Sizes

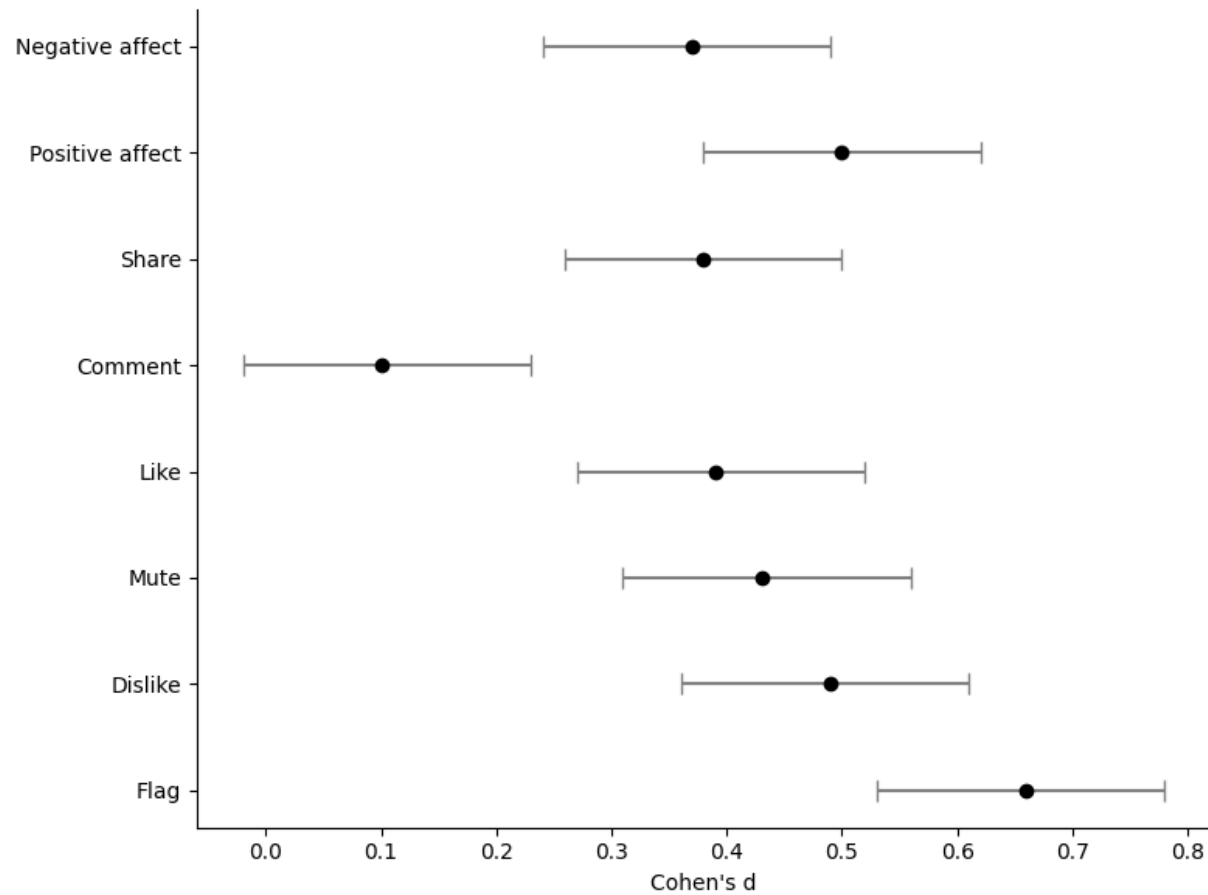

Supplement: S5 Appendix — (PDF) [file pone.0341907.s005.pdf]
